# Increasing efficiency and treatment volumes for sonolysis of per- and poly-fluorinated substances, applied to aqueous film-forming foam

**DOI:** 10.1016/j.ultsonch.2024.106866

**Published:** 2024-04-01

**Authors:** Tim Sidnell, Jake Hurst, Judy Lee, Madeleine J. Bussemaker

**Affiliations:** a School of Chemistry and Chemical Engineering, University of Surrey, Guildford, Surrey, GU2 7XH, United Kingdom; b ARCADIS, 1 Whitehall Riverside, Leeds, LS1 4BN, UK, United Kingdom

**Keywords:** PFAS, Sonolysis, Scale up, Ultrasonic degradation, Parallel transducers, Lightwater

## Abstract

Sonolysis of per- and polyfluoroalkyl substances (PFAS) has recently matured to field studies, treating real world contamination. However, efficient sonolysis reactor designs are poorly researched. Moreover, the variety and complexity of PFAS pollution slows reactor optimisation and scale-up. In this work, the defluorination of 10.0 mg/L aqueous perfluorooctane sulfonic acid (PFOS) was used as a model metric for the optimisation of; reactor volume (0.6 or 1.4 L), power density (100 – 350 W L^-1^), number of modular reactors (1–3), and liquid height (56.7 – 340 mm). Note, the ultrasonic frequency (410 kHz) and flow rate (214.2 ml min^−1^) was optimised in this reactor previously. Peak PFOS defluorination rate (3.40 μmolL^-1^ min^−1^) occurred at 141.8 mm, in a 0.6 L reactor, under 200 W L^–1^ ultrasound. Increasing the number of transducers connected in parallel to one amplifier was able to increase treatment efficiency from 78.6 to 191.8 μmol kWh^−1^. The model was validated using legacy aqueous film forming foam (AFFF, 3 M FC-602 Lightwater) at different dilutions (×5, ×10, ×20 and ×100). Dilution played a role in AFFF sonolysis efficiency with optimal PFAS sonolysis rate (4.28 μmol L^-1^ min^−1^) at 20 × dilution. Overall AFFF was effectively modelled with a synthetic PFOS solution, attributed to limited matrix effects in AFFF sonolysis and high PFAS concentration (0.18–1.83 g L^-1^) dominated by PFOS (0.15 – 1.53 g L^-1^).

## Highlights

• Liquid height had minimal effect on defluorination rate at constant power density.
• Power density was the defining factor to increase defluorination rate.
• Use of multiple reactors/transducers in parallel in a modular design demonstrated.
• 3 M Lightwater defluorination optimised at 20 × pre-sonolysis dilution.

## 1 Introduction

Per- and polyfluoroalkyl substance (PFAS) pollution is ubiquitous throughout the world’s flora, fauna, soils, and water [1]. Known to bioaccumulate in humans [2], leading to cancers and several other illnesses [3], three previously common PFAS and related compounds are now banned for all but a few uses as detailed in the Stockholm Convention [4]. The C_n_F_2n+1_- moiety in PFAS compounds imparts heat- and chemical resistance, as well as hydro- and lipo-phobicity [5], [6]. While useful in many applications, these properties hamper PFAS capture and destruction [7], [8]. As such, immense research efforts currently concern separative and destructive technologies for PFAS in several media, across an extensive concentration range [9], [10], [11], [12], [13], [14], [15], [16], [17], [18]. Sonolysis is one promising technology for PFAS degradation [9], [19], [20], and other pollutants. In sonolysis, longitudinal ultrasonic pressure waves generate and manipulate gas bubbles within the contaminated liquid, causing the bubbles to oscillate and expand due to rectified diffusion of gas into the cavity [21], [22]. Since many PFAS are surfactants, they accumulate at the bubble air–water interface [23], [24]. At a critical size, the bubbles implode, generating high temperatures (≥800 K), high pressures (several hundred bar) [25], radical species (OH•, O_2_^–^) [26], and aqueous electrons [27], [28], [29], [30], [31], all of which may contribute to PFAS sonolysis [9], [20]. The process mineralises PFAS into relatively harmless inorganics; (H^+^ or H_2_) [9], CO, CO_2_ [32], F^-^, and SO_4_^2-^ [9], [20], [23].

Sonolysis for PFAS degradation has broad applicability and PFAS sonolysis has been reviewed elsewhere [9], [19]. In general, sonolysis generates fewer shortened PFAS than competitor technologies, in exchange for moderately high energy costs [9] and moderate treatment times (up to several mg L^-1^ h^-1^ treatment rates) [20], [23]. The technology is effective for all PFAS structures tested to date, and is proven in; pure aqueous solutions [20], [23], [24], [26], [32], [33], [34], [35], [36], [37], groundwater [38], landfill leachate [39], aqueous film-forming foams (AFFFs) [40], [41], [42], soil slurry [43], and investigation derived waste (IDW) [44]. Only one work has reported failed PFAS sonolysis in raw sewage at low frequency [45]. However high frequency ultrasound (200–1000 kHz) is known to be more effective for PFAS sonolysis [9].

Sonochemical reactor designs (especially for water treatment) were not well studied until the 1990s [46], [47] and computational sonochemistry modelling was previously limited to single bubble systems [48], not typically used for sonolysis [35], [41], [49]. Industrial ultrasound usage has focused on mixing, emulsifying, and crystallisation in food [50], pharmaceuticals [51], and dyes [47]. These applications use low-frequencies (<100 kHz) [52], [53]), which have limited degradation potential [35], [41], [49], [54]. Sonication has also been termed an “auxiliary” treatment [52], likely due to its higher power demands than traditional waste water treatment plants (WWTP). However, since traditional technologies cannot degrade PFAS [10], [11], [14], [18], [55], relative power requirements are of less concern.

**Fig. 1:**
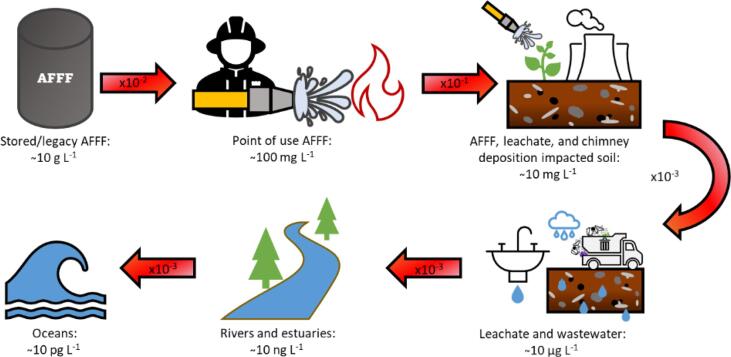
Approximate order of magnitude concentrations seen in various PFAS-containing media [66], [69], [70], [71], [72], [73].

PFAS sonolysis has moved from laboratory scale (<0.5 L) to bench scales (12–91 L) [35], [37], [41], [44], and recently been demonstrated for portable, on-site treatment of PFAS contaminated groundwater [56]. Reactor designs are presented without justification or experimentation on configuration. A large scale, general purpose, PFAS sonolysis reactor is challenged by the variable nature of PFAS pollution, which encompasses several matrix types, numerous co-contaminant effects, and concentrations spanning up to twelve orders of magnitude (Fig. 1). Further, with decreasing concentration, the volume to be treated generally increases, from a few thousand tonnes of stockpiled AFFF [57] to entire oceans. Additionally, over 9,000 PFAS structures exist [58], [59], with different removal rates, depending on the technology used, their structure, and concentration [14], [24], [60]. Thus the challenge of implementing sonolysis is for efficient scale-up that can encompass the broad characteristics of PFAS contaminations.

This work experimentally considers, for the first time the scale-up effects of increasing ultrasonic power, multiple reactors in parallel, reactor headspace, and liquid height applied to perfluorooctane sulfonic acid (PFOS). PFOS was chosen as a model compound because it represents the dominant PFAS found in many environmental [61], [62], WWTP [63], [64], and AFFF [65], [66] samples, and is highly regulated as a persistent organic pollutant [67]. Further, PFOS is sonolysed slower than several other common PFAS, including; perfluorooctanoic acid (PFOA), Perfluorohexanoic acid (PFHxA), and 6:2 Fluorotelomer surfactant (6:2 FTS) [9], [23], [33], [43], [68]. The defluorination rate of PFOS was used as a model metric for optimisation, owing to its simple and rapid measurement and prior correlation with complete PFOS mineralisation [20]. A high concentration of PFOS (10 mg L^-1^) was used during the modelling phase since this represents a similar concentration to those seen in diluted AFFFs [40], [41], [42].

## 2 Materials and methods

The chemicals, equipment, reactor configurations, and methods used here were similar to those in previous research [74], [75]. Unless otherwise stated ≥ 3 repeats were completed for each experiment, such that averages, and standard deviations are presented. Similarly, sonoluminescence (SL) images presented are representative of three replicates.

### 2.1 Materials

98 % potassium heptadecafluorooctanesulfonic acid (PFOS), ≥99.9 % HPLC-grade methanol, and analytical standard 0.1 M sodium fluoride (NaF) were purchased from Sigma-Aldrich®. Total ionic strength adjustor I (ISA) was purchased from Cole-Parmer (contains by weight: acetic acid 1.4 %, sodium acetate 8.2 %, sodium chloride 5.8 % and trans‐1,2‐diamino cyclohexane tetra acetic acid 0.4 % in deionised water). pH buffer tablets (4.0, 7.0, and 9.2) were purchased from VWR Chemicals, containing: 1,2-benzenedicarboxylic acid, monopotassium salt (pH 4); disodium orthophosphate heptahydrate, sodium chloride, dipotassium phosphate (pH 7); and borates, sodium salts, anhydrous, sodium chloride, (pH 9.2). Distilled water (Milli-Q) was provided by an Elix Essential 3 (UV) Type 2 device operating at 18.2 MΩ cm.

### 2.2 Reactor design and operation

**Fig. 2:**
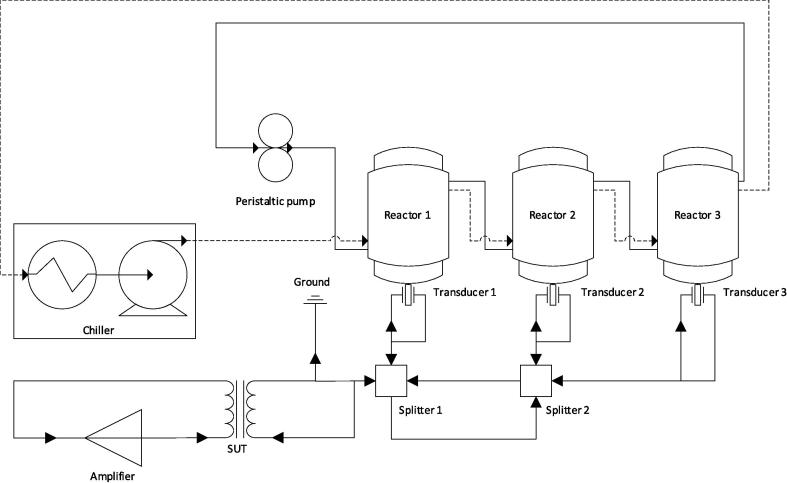
Electronic, process fluid, and CW connections in the reactor system.

Solutions were sonicated in 1–3 × 0.6 L or 1.4 L glass reactors, with an internal diameter of 6.7 cm (Fig. S1, supplementary information). Due to a transducer mounting flange, the lower third of the 0.6 L reactor was not jacketed and therefore not temperature controlled. Hence, the liquid volume was kept above 0.2 L. Similarly, in the 0.6 and 1.4 L reactors, the volume was kept below 0.5 and 1.3 L, respectively, to prevent interference between the lid and waterspout formed under sonication. 10.0 ± 0.8 °C cooling water (CW) was supplied to the reactors using a 20 L Cole-Palmer PolyScience MX recirculating chiller (Fig. 2). Temperatures recorded during experimentation are shown in Table S1-S2. To avoid interference by the thermocouple, the reactor temperature was not directly controlled, but was measured when the ultrasound was silenced for sampling. CW was pre-cooled prior to any experiments or measurements of power consumption. In some trials, the reactors were connected in series to a recirculating peristaltic pump, operating at 214.2 ml min^−1^ (Fig. 2), optimised in previous research [76]. Here, an additional 20 ml of fluid was added per reactor to account for the liquid height loss due to fluid entering the pump tubing. Power consumption by the amplifier, chiller, and pump were measured to ± 0.01 kWh using a UK version Prodigit Electronics Co. Ltd. Model 2000MU Plug-in power monitor [77].

A Honda electronics Co. LTD. transducer with 400 kHz nominal frequency was attached to each reactor base. Note, 400 kHz was the optimum frequency for PFOS degradation in our prior work with this equipment [20]. However, the transducer used in this work operated best (with lowest reflected power) at 410 kHz, giving optimised treatment efficiency and a reduced risk of equipment damage. The transducers comprised a 5.0 cm diameter PZT piezo-electric ceramic disk glued to a 10.0 cm diameter stainless steel plate. An AB-type AG 1006 T&C Power Conversion amplifier (amp) supplied power to the transducers (the load). Multiple transducers (and hence reactors) received the signal in parallel via one or two RF splitters (Fig. 2). The amp consumes a constant power which is modulated at the output using a variable resistor and heat sink [78], [79], providing 0–300 W. The amp and load impedances were matched via a T&C Power Conversion RF impedance step-up transformer (SUT). For parallel transducers, the load impedance (Z_PT_) reduced with added transducers, according to Eq. (1) (derivation given in supplementary information section C).

(1) $$Z_{\mathrm{PT}}=\frac{Z_{i}}{n}$$

Where n is the number of parallel transducers and $Z_{i}$ is the load impedance of a single transducer. For parallel transducers, the amp impedance exceeded the load impedance, so the SUT input and output were swapped to operate as a step-down transformer. Static was discharged through a 10 kΩ resistor connected between the step-up/down transformer and the load. The forward power $\left(P_{F},W\right)$ sent from the amplifier was automatically modulated against reflected power $\left(P_{R},W\right)$ (according to Eq. (2) to provide constant load power $\left(P_{L},W\right)$. Frequency was tuned to minimise reflected power, normally in the order of less than 10 % of forward power. For n transducers, the load power was set to n times the desired load for each transducer since the signal was near-evenly split among transducers.

(2) $$P_{F}=\;P_{L}+P_{R}$$

Load power density $\left({\mathrm{PD}}_{L},WL^{-1}\right)$ [19], [33], [36], [80] and load power intensity $\left({\mathrm{PI}}_{L},W{\mathrm{cm}}^{-2}\right)$ were calculated using Eq. (3) and Eq. (4), respectively.

(3) $${\mathrm{PD}}_{L}=\frac{P_{L}}{V}$$

(4) $${\mathrm{PI}}_{L}=\frac{P_{L}}{A_{t}}$$

Where: V is the liquid volume (L) and $A_{t}$ is the transducer plate-liquid contact area (35.26 cm^2^).

Defluorination efficiency was used to compare efficiency of various treatments, calculated based on the total power drawn by the chiller and amplifier (Eq. (5).

(5) $$\eta =\frac{F_{\mathrm{TOT}}}{{\mathrm{PW}}_{C}+{\mathrm{PW}}_{A}+{\mathrm{PW}}_{P}}$$

Where: $F_{\mathrm{TOT}}$ is the total fluoride released over the course of the experiment (μmol); and ${\mathrm{PW}}_{C}$, ${\mathrm{PW}}_{A}$ and ${\mathrm{PW}}_{P}$ are the power drawn over the course of the experiment (kWh) by the chiller, amplifier and pump, respectively.

### 2.3 Optimisation procedure and AFFF sonolysis

The parameters optimised using the PFOS model were; reactor volume (0.6 or 1.4 L), power density (100–350 W L^-1^), power intensity (1.13–3.97 W cm^−2^), number of parallel reactors (1–3), and liquid height (56.7–141.8 mm). Unless otherwise stated defluorination rate is reported from 30 min of sonication based on pseudo-zero order kinetics, demonstrated in past work [20]. The AFFF was sonicated using the optimised conditions using four different dilutions such that defluorination and PFAS removal rates could be compared. Adjustments to the AFFF sonolysis approach were required to incorporate flow-though conditions, at 214.2 ml min^−1^ recirculation rate (previously optimised [76]); 3 × 500 ml was used with 270 W L^-1^ ultrasound at 410 kHz, 3 × parallel reactors. AFFF dilutions included 5×, 10×, 20×, and 100 ×. Defluorination rates were calculated over 2 h using 30 min time intervals (5×, 20 × ), and for 8 h using 1 h time intervals (10×, 100 × ). Longer studies were conducted to inform defluorination order at two ‘high’ and ‘low’ dilutions.

### 2.4 Sampling and cleaning

Sonication time for optimisation experiments was 30 min with sampling every 5 min to inform defluorination rates. In AFFF, sonication was conducted over 2 or 8 h time periods with sampling every 30 min due to the higher PFAS concentration. When assessing the defluorination rate via ion selective electrode (Section 2.4.2), a 2.00 ml sample was gathered with a 1.00–5.00 ml micropipette with 5.00 ml disposable polypropylene tips. Between experiments, all containers were flushed with methanol in triplicate, to desorb any adsorbed PFAS [81], and the thermocouple and electrode were both washed using Milli-Q water and dried using lint-free tissue.

### 2.5 Reaction characterisation

#### 2.5.1 Fluoride release

F^-^ concentration was measured using a Cole-Parmer Combination Fluoride Ion Selective Electrode (or Probe) connected to a conductivity meter, as per prior work [20], [82]. The F^-^ probe was calibrated by adding 0.1 M aqueous NaF solution to the sample of PFOS solution or AFFF, then successively adding sample to create a dilution series. 2 ml of sample was magnetically stirred at 100 rpm and temperature controlled at 25.0 ± 0.05 °C using a heated magnetic stirrer and polypropylene coated stirrer bar. The probe was inserted into the solution, at constant depth, and 0.2 ml of ISA added. Sample conductivity (σ, mV) was recorded once the conductivity meter indicated a stable reading. This procedure was applied to samples from the reactor and the conductivity converted to [F^-^] using the derived correlations (see Fig. S2). Below 50 µM L^-1^, the [F^-^] measurement was unquantifiable (see Fig. S2) in the AFFF, possibly due to pH values and organics co-contaminants outside the recommended range [82].

#### 2.5.2 Sonoluminescence (SL) / sonochemiluminescence (SCL)

SL/SCL emissions were captured using an ANDOR iXon3 EMCCD camera operated at − 70 °C placed adjacent to the reactor, in a light-sealed box, within a darkened room at 19.0 °C. SL of AFFF, Milli-Q water and PFOS solution were captured with an EM gain of 50 and exposure time of 15.0–60.0 s using ANDOR software. SCL emissions were similarly assessed by sonicating a solution of 0.1 M NaOH and 1.0 mM luminol using an EM gain of 4 and exposure time of 2 s. Sonication started 30 s prior to image capture, to allow bubble populations and light emissions to stabilise [74], [83]. SL emissions were not quantified in AFFF due to the varied exposure time between settings, used to obtain images. The images instead demonstrate, qualitatively, where sonochemical/thermal activity occurs in the reactor.

#### 2.5.3 Iodide dosimetry

To indicate the relative concentration of radical species formed during sonication, Hart and Henglein’s dosimetry method was used [84]. 0.1 M Potassium iodide (KI) and 0.5 mM ammonium molybdate tetrahydrate solution was sonicated with 10.0 °C cooling water supplied, and 2.00 ml samples taken every 5 min. The ammonium molybdate catalyses hydrogen peroxide’s reaction with iodide, which is otherwise too slow to detect H_2_O_2_ formation. Using an Evolution 201 UV–vis spectrometer, the absorbance of 350 nm light by the samples was measured using a 1.0 cm wide quartz cuvette. However, the solution absorbance saturated within a few minutes of sonication due to high levels of I_3_^-^ production. Therefore, the 2 ml samples were diluted 5.0x to obtain differentiable results. According to the Beer-Lambert law, modified to account for the dilution, the concentration of I_3_^-^ ions was calculated from Eq. (6).

(6) $$C=\;D\frac{A}{\varepsilon l}$$

Where; A is the absorbance of the sample (-), ε is the molar absorptivity coefficient of I_3_^-^ ions (26,303 L mol^−1^ cm^−1^) [73], C is the concentration of I_3_^-^ ions (mols L^-1^), D is the dilution factor (5.0, -), and l is the length of light path (cuvette width = 1 cm). It should be noted that the use of this method in air saturated systems can lead to inaccurate *absolute* radical concentration measurements since sonolysis of N_2_ and O_2_ forms nitric acid which can oxidise KI [21], [85], [86]. Hence, this method was used to investigate the *relative* radical production between different heights.

#### 2.5.4 Calorimetry

Calorimetric power ($P_{\mathrm{Cal}}$) was calculated using a previously derived method [87], [69] which was modified to account for heat losses through the reactor walls (Eq. (7) [76].

(7) $$P_{\mathrm{Cal}}=mC_{p}\frac{\mathrm{dT}}{\mathrm{dt}}+\;{\left(P_{\mathrm{Cool}}\right|}_{\overset{¯}{T}}$$

Where; $P_{\mathrm{Cal}}$ is the calorimetric power (Watts), m is the mass of water (g), $C_{p}$ is the specific heat capacity of water (taken as constant, 4.18 J g^−1^ K^−1^), $\frac{\mathrm{dT}}{\mathrm{dt}}$ is the temperature (T) change during time $t_{0}$ to $t_{1}$, = $\frac{T_{1}-T_{0}}{t_{1}-t_{0}}$ (K/s), and ${\left(P_{\mathrm{Cool}}\right|}_{\overset{¯}{T}}\left(=mC_{p}\frac{dT_{\mathrm{Cool}}}{dt_{\mathrm{Cool}}}\right)$ is the cooling power (W) i.e., the rate of heat loss through the walls of reactor evaluated at the average temperature, $\overset{¯}{T}=\frac{T_{1}-T_{0}}{2}$. $P_{\mathrm{Cool}}$ varied depending on the solution temperature and volume, so was determined by monitoring the temperature change over time ($\frac{dT_{\mathrm{Cool}}}{dt_{\mathrm{Cool}}}$) of a given volume of 80.0 °C water added to the reactor.

## 3 Results and discussion

### 3.1 Reactor optimisation using PFOS defluorination

#### 3.1.1 Power density/Intensity

**Fig. 3:**
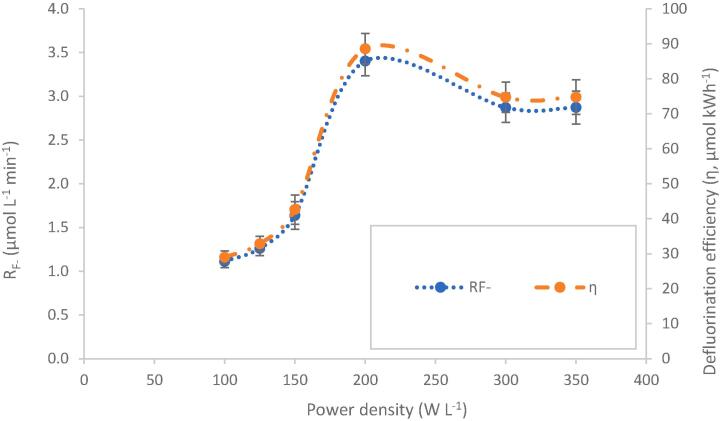
Effect of increasing load power/power density on the defluorination rate and defluorination efficiency (η, µmol kWh^-1^) of a 400 ml solution of 10.0 mg L^-1^ PFOS (0.6 L reactor) using 410 kHz ultrasound, with cooling water supplied.

10 mg L^-1^ PFOS was sonicated for 30 min sonication under 410 kHz ultrasound, in the 0.6 L reactor, and load powers were varied from 40 to 140 W, giving load power densities (${\mathrm{PD}}_{L}$) of 100–350 W L^-1^. The power densities are equivalent to power intensities (${\mathrm{PI}}_{L}$) of 1.13–3.97 W cm^−2^ which in this case are directly proportional to power density since volume is constant. Discussions here are made with reference to power density. Since the solution volume and power consumption by both the chiller and amplifier were constant, defluorination efficiency (η, μmol kWh^−1^) followed the same trend as the fluoride release rate, included here for later comparison (Fig. 3). An increase in applied power from 40 to 80 W (100 – 200 W L^-1^), resulted in a near-exponential increase in defluorination rate (R_F-_) from 1.11 to 3.40 μmol L^-1^ min^−1^ (Fig. 3). However, above 200 W L^-1^ the rate reduced and plateaued at ≈2.87 μmol L^-1^ min^−1^. The inflection point at 200 W L^-1^ suggests an optimal setting resulting from competing effects in the reactor. In prior work, PFAS reaction rates also plateaued at particularly high or low power densities, creating an overall S-curve for reaction rate vs power density [33], [88].

The observed effects may be due to temperature effects, changes to bubble symmetry and/or decoupling. The temperature increases at 300 W L^-1^ and 350 W L^-1^, where rates plateaued, were ∼ 20 °C (reaching a maximum of 43 °C) versus ∼ 5 °C at lower power density (Table S1). Temperatures can impact the speed of sound, water evaporation into the bubble, gas solubility and bubble size, all of which impact collapse intensity and temperature of collapse [25]. Previous work at 500 kHz indicated a maximum efficiency of degradation of 4-chlorophenol/sonochemical oxidation at around 40 °C [89]. PFOS, however, is not oxidised by hydroxyl radicals, and relies on other mechanisms that occur during bubble collapse [9]. A temperature effect does not fully explain the similar S-curve response reported when temperature was controlled in other PFAS systems [33]. Reports of power density effects on PFOS degradation at 575 kHz attribute the plateau to an increase in asymmetric bubble collapse, reducing sonochemical effects [88]. A similar trend was observed at 1 MHz for potassium iodide dosimetry, using a plate-liquid contact area of 38.48 cm^2^, which showed a maximum reaction rate at 2.08 W cm^−1^ (80 W $P_{L}$), with ≈93 % decrease at 2.86 W cm^−2^ (110 W $P_{L}$) [90]. Here, the maximum R_F-_ observed at 200 W L^-1^ (80 W $P_{L}$) had a calorimetric power intensity of 2.27 W cm^−2^, after which the rate decreased. Gutierrez and Henglein [90] attributed the rapid rate loss to “decoupling”, i.e., the formation of large and chemically inactive bubbles near the transducer plate at sufficiently high power intensities, which also attenuate pressure waves before they traverse the full fluid volume [47], [90], [91], [92]. Loss of defluorination efficiency at higher power density, is therefore likely contributed to, in part, by decoupling. This will be discussed further in 3.1.3, 3.1.4.

#### 3.1.2 Comparison of small (0.6 L) and large (1.4 L) reactors

Using the same conditions as Section 3.1.1 and 80 W *P_L_* (200 W L^-1^ *PD_L_*), at a constant liquid height of 113.5 mm (0.4 L), with 10.0 °C CW supplied, different reactors were compared. The idea was to choose the most appropriate reactor for subsequent optimisation, and to enable an increase of height. The defluorination rate (R_F-_) achieved was ≈ 3.40 μmol L^-1^ min^−1^ (±5 %) in the 0.6 L reactor but 2.55 μmol L^-1^ min^−1^ (±1.2 %) in the 1.4 L reactor. Defluorination efficiency (η, μmol kWh^−1^) followed the same trend as previously observed in Section 3.1.1. (results not shown). The 67 % additional mass of reactor wall in the 1.4 L reactor likely attenuated a greater proportion of the sound wave [93] and hence lead to a reduced R_F-_. Optimisation was continued in the 0.6 L reactor.

#### 3.1.3 Liquid height and applied cooling

For any implementation of PFAS sonolysis, consideration of volume increases, and additional energy required for temperature control are key factors. Therefore, five liquid heights were tested (56.7, 70.9, 85.1, 113.5, and 141.8 mm, volumes of 0.20, 0.25, 0.30, 0.40 and 0.50 L respectively) in the 0.6 L reactor with and without cooling water. Frequency of 410 kHz was applied at power densities 100 and 200 W L^-1^. The power density was held constant by proportionally increasing $P_{L}$ with liquid height. Increasing $P_{L}$ relates to an increase in power intensity since power intensity is a ratio of $P_{L}$ to transducer surface area, which remained constant (Table S3). When applied, cooling water maintained the solution 24.7 ± 6.7 °C and 25.4 ± 6.5 °C for ${\mathrm{PD}}_{L}$ = 100 and 200 W L^-1^, respectively (Table S2). [F^-^] measurements every five minutes revealed a zero-order rate of PFAS degradation/ F^-^ formation (0.9939 < R^2^ < 0.9993), consistent with previous results from similar conditions [20]. Note for every 1 M of PFOS mineralised 17 M of fluoride are produced, here rates of defluorination are compared as the most reliable means to appreciate PFOS mineralization [9].

*Effect of applied cooling water*.

**Fig. 4:**
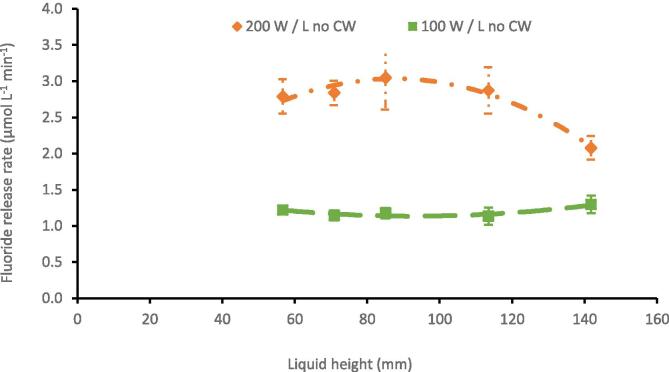
RF- from the sonolysis of 10.0 mg/L PFOS under 410 kHz ultrasound, in a 0.6 L reactor, at 100 W/L and 200 W/L load power, both without CW, at five liquid heights. Error bars represent the standard deviation of three repeats.

**Fig. 5:**
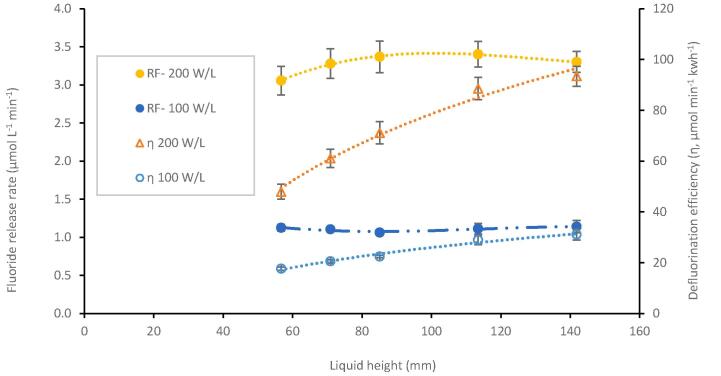
Defluorination rate (RF-) and defluorination efficiency (η) from the sonolysis of 10.0 mg/L PFOS under 410 kHz ultrasound, for 30 min, in a 0.6 L reactor, at 100 W/L and 200 W/L load power, at five liquid heights, with cooling water. Error bars represent the standard deviation of three repeats.

Cooling water had little impact on fluoride release rate at 100 W L^-1^, although increased the variability between experiments at both power inputs (Fig. 4, Fig. 5, see Fig. S3 for comparison). This could be due to the increased power density and dynamic bulk mixing exacerbating the effects of perturbations in external conditions i.e. the room temperature variation (21 ± 3 °C) resulted in higher variation in repeats that were slightly more/less aerated, warm, etc. This suggests an increased sensitivity to initial/external reactor conditions (ambient temperature, bubble size distributions, concentration, etc.), which could be problematic for industrial scale PFAS remediation. At 200 W L^-1^ there was a drop in defluorination rate (∼35 %) at 141.8 mm without cooling water. Differences in fluoride release rates at other liquid heights (56.7–113.5 mm) were not statistically significant.

Without cooling water, the solution temperature under 100 W L^-1^ ultrasound increased from room temperature by ∼ 23–25 °C across the different liquid heights (Table S2). Whereas at 200 W L^-1^, the temperature increased by ∼ 39–50 °C at different liquid heights (Table S2). Here, an approximate temperature increase above 30 °C from room temperature (up to > 60 °C) seems to negatively impact fluoride release rates, as previously reported in sonochemical oxidation at 500 kHz [89]. However more focussed temperature-controlled experiments in PFAS solutions would be required to confirm this. Note that temperature differences at 100 W L^-1^ had no clear impact on degradation for most points, hence, the observed rate plateau in Section 3.1.1 has likely contributions from other effects such as decoupling and/or bubble asymmetry. This will be discussed further in Section 3.1.4.

*Effect of liquid height*.

With cooling water, defluorination rates at different liquid heights i.e. constant power densities were not statistically different (Fig. 5). From average rates there was an ± 8 % and ± 11 % variation in defluorination rate under 100 W L^-1^, and 200 W L^-1^ (Fig. 5, Table S4). As liquid height increased, at each power density, power intensity is increased (Table S3). A plot of defluorination rate versus power intensity shows no correlation (Fig. S4) which indicates that power intensity does not significantly impact cavitation that supports PFOS sonolysis. Defluorination efficiency increased pseudo-linearly with increasing liquid height for both power densities (Fig. 5). (Note that defluorination efficiency was not significantly different in comparison of 113.5 and 141.8 mm at 200 W L^-1^_._) The reduced relative effect of height between 113.5 and 141.8 mm at 200 W L^-1^ on defluorination efficiency (with cooling water), suggests that physical effects of liquid height (i.e. strength of standing wave formation and attenuation [94]) were reduced with enhanced sound pressure [91], especially compared to other works [95], [96], [97] where power density was not constant. Decoupling may also explain the slight loss in defluorination rate at the highest liquid volume and power under 200 W L^-1^. Given more efficient treatment using multiple reactors (Section 3.1.5) and diminishing returns of using 141.8 mm, a liquid height of 113.5 mm was selected for use in the modular reactor design at 200 W L^-1^.

#### 3.1.4 Liquid height effects on SL, SCL, dosimetry and calorimetry

**Fig. 6:**
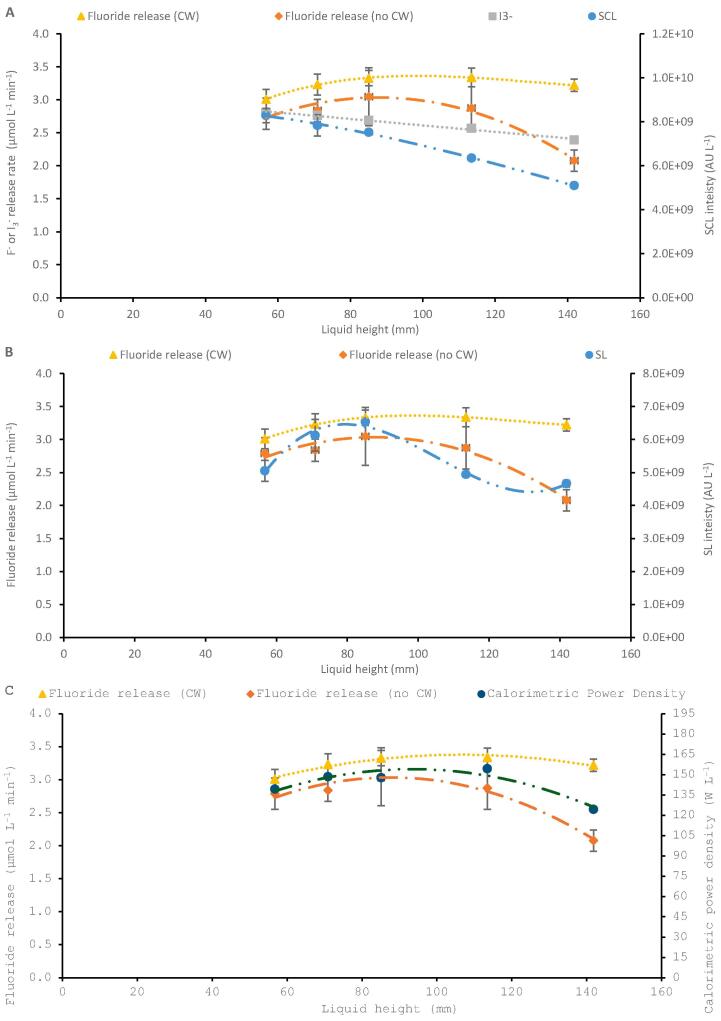
F- release rate of PFOS (both with and without CW) correlated with triiodide production rate and sonochemiluminescence (A), sonoluminescence (B), and calorimetric power density (C) across five liquid heights and load powers, at constant 200 W/L power density under 410 kHz ultrasound). For (A) and (B), SL and SCL are measured in arbitrary units (AU) per litre.

Correlation of SL, SCL, iodide dosimetry and calorimetry with PFOS defluorination rates were made to further elucidate the phenomenological effects of increasing liquid volume and constant power density of 200 W L^-1^. Note that all measurements are shown per litre of reaction fluid, for fair comparison to R_F-_. There was limited correlation between R_F-_ and the two sonochemical measurements (dosimetry and SCL), since R_F-_ increased then decreased with liquid height, while I_3_^-^ production and SCL intensity both decreased linearly (R^2^ = 0.9900 and 0.9899) (Fig. 6A). When comparing R_F-_, with and without CW, and SL intensity (Fig. 6B) there is a slight correlation (more so for R_F-_ without CW and SL) since all three data sets show an initial increase, followed by a decrease, in value with increasing liquid height (and absolute power/intensity). However, there is a much more significant (still imperfect) correlation with calorimetry and fluoride release rate, in both temperature controlled and uncontrolled cases (Fig. 6C). The lack of correlation with sonochemical activity is somewhat in contrast to our prior work in the same reactor [20], but which varied frequency, not power. However, the ${\mathrm{PI}}_{L}$ is up to 2.5 x that used previously [20], hence, there is likely some blend of sono-mechanical, sonochemical, and sono-thermal effects here which were not seen previously.

**Fig. 7:**
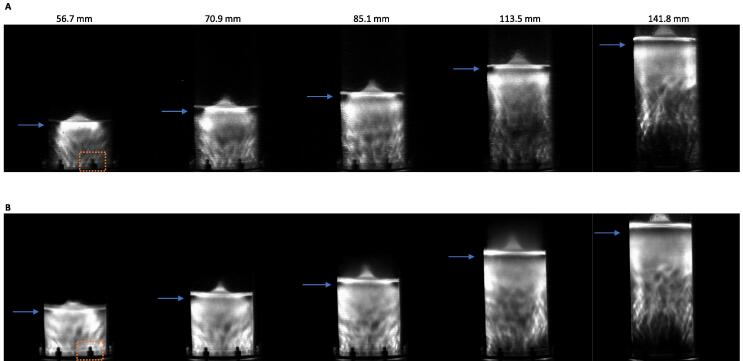
Representative sonoluminescence (A) and sonochemiluminescence (B) images (one of three repeats) for sonolysis of Milli-Q water using 410 kHz ultrasound at 200 W/L applied power, in liquid heights of 56.7, 70.9, 85.1, 113.5 and 141.8 mm, respectively. Note the bold shadows seen at the bottom of the photographs are due to screws and nuts which affix the transducer to the reactor base (examples shown with orange dotted box).

Selected SL and SCL images taken at each of the five liquid heights studied under 200 W L^-1^ are shown in Fig. 7A and B, respectively. Repeats of SL/SCL images and those taken under 100 W L^-1^ are shown in Fig. S5. Both SL and SCL image sets show bright regions of activity close to the liquid surface, which gradually shrink in width as power input/liquid height is increased, leading to the formation of, sonochemically inactive regions (dead zones) highlighted by the blue arrows (Fig. 7A and B). More significantly, however, are the large dead zones near the transducer. The SL images under 100 W L^-1^ (Fig. S4) show similar dark regions but across all liquid heights, suggesting that these are caused by the lack of power at each height and not overpowering causing decoupling. The SCL images at 100 W L^-1^, only show such dark regions at 113.5 and 141.8 mm, like those at 200 W L^-1^. The height of the dead zones under 100 W L^-1^ is constant, while the size of the active (bright) region increases as the liquid height is increased, showing the proportional growth of the active region down from the liquid surface (due to surface reflection) at higher powers. While under 200 W L^-1^, the height of the dead zone increases as the liquid height/power increases, attributed to enhancement of the decoupling. Similar observations were reported for the SL distribution in water sonicated using 142 kHz ultrasound [98] as well as those of thermal and SCL imaging of a cylindrical vessel with a similarly sized diameter (7 cm vs 6.7 cm here) and 490 kHz ultrasound driven at 29 W [97].

Loss of activity in high power regions may be due to decoupling and/or asymmetric collapse as discussed in Section 3.1.1. SL is known to form from relatively large and stable bubble collapses while SCL forms from smaller and more transient collapses [99]. High power likely caused transient collapse and thus the loss of SL at the transducer base and liquid surface, where power density is highest from initial transmission and wave reflection, respectively. This also suggests why the SL dead zones at 113.5 mm and 141.8 mm are darker than those for SCL. The more rapid decline in SL, SCL, and dosimetry with increasing absolute power also suggests that effective decoupling begins at a lower applied power for these metrics than for PFOS defluorination. Thus, the decoupling intensity limit for the ultrasound may vary depending on the specific activity being considered.

#### 3.1.5 Parallel reactors

**Fig. 8:**
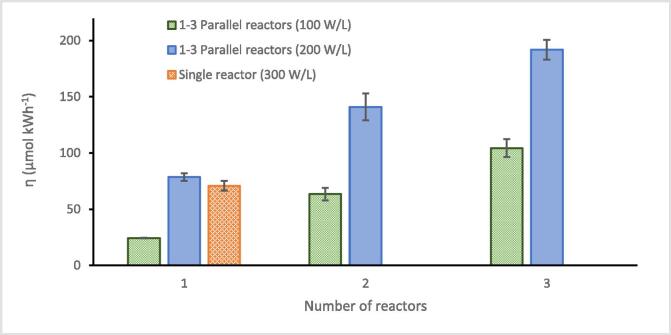
Defluorination efficiency (η) during 30-minute sonolysis of 10.0 mg/L PFOS under 410 kHz ultrasound, at 40–120 W per transducer (100–300 W L^-1^) load power, for 1–3 parallel transducers with 10.0 °C CW supplied. Error bars represent the standard deviation of three repeats.

Since reaction rates/efficiency were not enhanced above 80 W in a single reactor (Section 3.1.1), the use of multiple parallel reactors was used to reduce decoupling effects by spreading the applied power across a greater total plate area. Defluorination rates are reported using 1, 2 and 3 reactors (Table 1). Temperature increases in individual parallel reactors were within the same order as individual reactors (Table S1). Where more than one reactor is used the defluorination rate represents an average of the 2 or 3 reactors, and relevant experimental repeats. The zero-order defluorination rate had a maximum variation (i.e. difference in defluorination rates) of 48 % when applying 40 W per reactor (100 W L^-1^, increasing the number of reactors from 1 to 3, Table 2). However, at 200 W L^-1^ the rate variation (difference in defluorination rates) between 1 and 3 reactors was only 3.7 % and the maximum variation of rate 11 % (comparing 1 and 2 reactors). The rate variation was likely because the second and third transducers had resonant frequencies slightly above/below 410 kHz and thus had lower power conversion efficiencies. The 120 W used in the 300 W L^-1^ single reactor condition was more efficiently used when spread across three parallel reactors (Fig. 5). The highest defluorination efficiency of our trials was achieved using 80 W per reactor (200 W L^-1^) with three parallel reactors (Fig. 8). $P_{R}$ reduced with the number of transducers added (Table 1), which is consistent with the reduction of the total load impedance [79] (Eq. (1) and Section C of Supplementary) and improved impedance matching with the step-down transformer. The slightly increased power consumption under multiple transducers may be explained by a longer sampling time with more reactors since during sampling, the amplifier output was set to 0 W but remained on, and power was drawn. Therefore, slightly higher electricity consumption occurred with more reactors in consideration of sampling time.

**Table 1:** Power data (W) from a single amplifier and defluorination rate during 30-minute sonication of 10.0 mg/L PFOS in 1x, 2x, and 3x reactors containing 400 ml solution under 410 kHz ultrasound at 100–300 W/L_._

${\mathrm{PD}}_{L}$(W L^-1^)	${\mathrm{PI}}_{L}$(W cm^−2^)	No. of *trans*-ducers	$P_{L}$(Amplifier)	$P_{R}$(Amplifier)	$P_{F}$(Amplifier)	$P_{L}$ per transducer	Amplifier consumption (kWh)	Defluorination rate (μmol L^-1^ min^−1^)	Standard deviation
100	1.13	1	40	12.5 ± 0.50	52.5	40	0.28 ± 0.00	1.137	0.012
		2	80	2.0 ± 0.50	82.0	40	0.28 ± 0.00	1.480	0.127
		3	120	1.5 ± 0.50	121.5	40	0.29 ± 0.02	1.681	0.129
			Rate variation between 1 and 3 reactors						48 %
200	2.27	1	80	30.7 ± 0.33	110.7	80	0.26 ± 0.01	3.402	0.169
		2	160	2.0 ± 0.50	162.0	80	0.26 ± 0.03	2.997	0.251
		3	240	1.5 ± 0.50	241.5	80	0.31 ± 0.03	3.277	0.151
			Rate variation between 1 and 3 reactors						3.7 %
300	3.40	1	120	38.6 ± 1.20	158.6	120	0.28 ± 0.00	2.715	0.168

The optimum 3x 80 W arrangement utilised 80 % of the amplifier output, thus, a more powerful amplifier and greater number of transducers could augment efficiency. In this case the optimum efficiency was 192 ± 9 μmol kWh^−1^. Use of multiple transducers in a single reactor is established for low frequency applications [93] and has been investigated for large scale PFAS sonolysis [35], [41]. However, a study on the effects of increasing transducers/reactor numbers, has not been found to date.

### 3.2 AFFF sonolysis

AFFF of various dilutions (5×, 10×, 20×, and 100 × ) were sonicated based on optimal conditions from this work (Section 3.1) and the preceding publication [76] with slight modifications to allow flow through (see Section 2.3). In past work we found that flow could slightly enhance defluorination rates, and since this is advantageous for industrial implementation, these combined settings were tested for the real waste treatment [76]. Zero order defluorination rates were observed over the complete range of times studied: 2 h for 5 × and 20 × and 8 h for 10 × and 100× (see Table S5). Zero order defluorination rates, indicates that PFAS degradation within the AFFF was limited by bubble population / dynamics rather than the PFAS concentration at all dilutions [9].

The optimal defluorination rate (4.28 μmol L^-1^ min^−1^) was at 20 × dilution. Although there is insufficient information between 20 × and 100 × dilution to know whether this is a true optimum, and the processing of higher concentrations at 10x (which was not significantly lower than 20x) may be beneficial for overall treatment required. In prior studies [44], [40], [41], [42], AFFF concentrates were pre-treated by dilution with water 10 to 50,000-fold prior to sonication, likely due to the viscosity of the concentrate preventing direct sonication. Note that the viscosity of AFFF is 1950 mPaS compared to 0.89 mPas for water at room temperature. Since a correlation exists between PFAS concentration and sonolysis rate [26], [34], [36], [37], [68], [40], an optimum AFFF dilution likely minimises viscosity and maximises PFAS concentration. Prior works reported optimum dilution factors which depended on the AFFF brand, ultrasound frequency, and measured rate (PFAS degradation, defluorination, or SO_4_^2-^ release) [9]. Hence, the rates found here are likely specific to defluorination of 3 M Lightwater FC-600 (1991) and the experimental set-up. R_F-_ values of 10 mg L^-1^ PFOS and diluted AFFF were within the same order of magnitude, likely due to the zero-order sonolysis kinetics of PFAS concentrations exceeding 1.45–19.5 mg L^-1^ [9], [20] (here 10–3,600 mg L^-1^) and the AFFF’s PFAS composition being dominated by PFOS (82.4 % by mass, [100]). The second most prominent PFAS in the AFFF was PFHxS (9.4 %), which also has a similar structure and sonolysis rate to PFOS [24], [26], [33]. 13 other PFAS represented just 8.2 % of the AFFF PFAS composition [100].

**Fig. 9:**
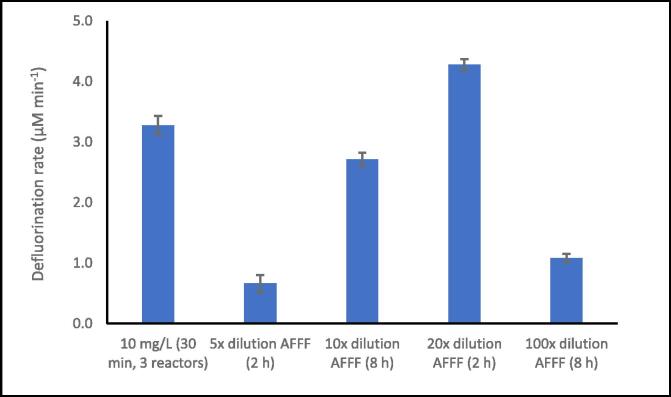
Defluorination rates from various concentrations of 3 M Lightwater compared to PFOS. Flow-though conditions, at 214.2 ml min^-1^ recirculation rate; 3 x 500 ml was used with 270 W L^-1^ ultrasound at 410 kHz, 3 x parallel reactors. Confidence intervals for AFFF displayed are based on standard error of regression slope. Experiments were conducted at least in duplicate if not triplicate.

**Fig. 10:**
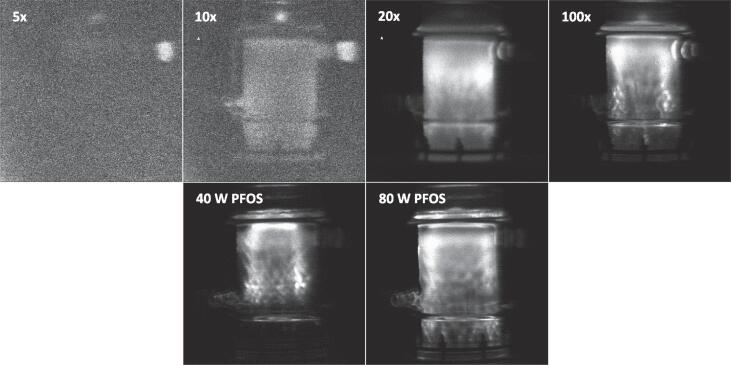
SL images of diluted 3 M Lightwater (above) and 10.0 mg/L PFOS (below). The images were captured using an EM gain of 50 s, exposure time of 60 s, (5×, 10×, and 20 × ) or 15 s (100 × and both PFOS images). The ultrasound was set to 80 W and 410 kHz for all bar the “40 W PFOS” image.

The dependence of AFFF defluorination rate on dilution (Fig. 9) indicates matrix effects impact sonolysis efficiency since PFAS concentrations exceed the previously measured zero-order rate limit [26], [34], [36], [37], [68], [40]. To further understand dilution effects, SL images (Fig. 10) were used to compare SL activity in different dilutions compared to PFOS in water. Increasing dilution from 5 to 20×, increased SL activity throughout the reactor. Note that 5x and 10x dilutions were too grainy for meaningful interpretation due to low SL intensity. Since SL is an indicator of bubble collapse intensity [101], this suggest more and larger sonochemically active bubbles were formed with increased dilution (and reduced viscosity). Therefore, thinning of the initially viscous concentrate, to allow more sonochemically active bubbles to form, may explain the rate increase from 5 to 20x dilution.

However, an explanation for the rate decrease from 20 to 100x dilution is less obvious. The solubility of certain co-contaminants (and hence their impact on bubble dynamics) may change as the fraction of water increases. While Lightwater’s composition varies by year of manufacture [66], it can contain F^–^ and SO_4_^2-^ (μmol L^-1^) [42], which likely have slightly negative impacts on defluorination at much higher concentrations (mmol L^-1^) [38]. VOCs (such as methyl *tert*-butyl ether, MTBE) in the mol L^-1^ range can also reduce sonolysis rates, due to bubble collapse temperature quenching [39]. A common co-organic in AFFFs is butyl-carbitol, which represents around 12 % of the foam composition [40]. However, after 100 × dilution, this percentage would be < 1 % (mmol L^-1^) and, given butyl-carbitol’s vapour pressure (4 Pa at 20° C [102]) relative to MTBE (33,000 Pa at 25 °C [103]), it is unlikely to have caused significantly negative rate effects. It is possible that the PFAS kinetic transition concentration is different in diluted AFFFs, but further work is required to prove this.

## 4 Conclusions

Approaches to scale-up PFAS pollution sonolysis were modelled using PFOS defluorination and validated using real world wastes. Under two power densities, (100 and 200 W L^-1^) liquid height had limited impact on rates compared to power density. Matching of the reactor and fluid volumes optimised rates, likely due to increased sonic energy-wall attenuation in excessively large reactors. The use of cooling water to control temperature, decreased overall treatment efficiency due to reduced total power but in most cases but increase reaction variability. SL, SCL images supported the hypothesis that at higher power densities, decoupling or loss of activity occurred that likely contributed to reduced defluorination efficiency. Multiple parallel reactors boosted treatment efficiency, due to minimised reflected power and greater utilisation of the amplifier output. PFOS was a valid model for PFAS defluorination in diluted 3 M Lightwater AFFF. For the first time, sonoluminescence images were captured in diluted AFFF, showing bubble quenching effects at low dilutions.

## CRediT authorship contribution statement

**Tim Sidnell:** Writing – review & editing, Writing – original draft, Methodology, Investigation, Formal analysis, Conceptualization, Data curation. **Jake Hurst:** Supervision, Resources, Conceptualization. **Judy Lee:** Writing – review & editing, Supervision. **Madeleine J. Bussemaker:** Writing – review & editing, Writing – original draft, Supervision, Project administration, Formal analysis, Conceptualization, Resources.

## Declaration of competing interest

The authors declare the following financial interests/personal relationships which may be considered as potential competing interests: Madeleine Bussemaker reports financial support was provided by Arcadis. Jake Hurst reports a relationship with Arcadis that includes: employment. If there are other authors, they declare that they have no known competing financial interests or personal relationships that could have appeared to influence the work reported in this paper.
