# Supplementary material for: Increasing efficiency and treatment volumes for sonolysis of per- and poly-fluorinated substances, applied to aqueous film-forming foam
Source: Ultrason Sonochem. 2024 Apr 1;105:106866. doi: 10.1016/j.ultsonch.2024.106866 (PMC11026841; doi:10.1016/j.ultsonch.2024.106866)
Supplement: Supplementary data 1 [file mmc1.docx]

**Increasing efficiency and treatment volumes for sonolysis of per- and poly-fluorinated substances, applied to aqueous fire fighting foam**

*Tim Sidnell^a^, Jake Hurst^b^, Judy Lee^a^, Madeleine J. Bussemaker*^a^*

*^a^ School of Chemistry and Chemical Engineering, University of Surrey, Guildford, Surrey, GU2 7XH, United Kingdom.*

*^b^ ARCADIS, 1 Whitehall Riverside, Leeds, LS1 4BN, UK, United Kingdom*

*^*^e-mail:* [m.bussemaker@surrey.ac.uk](mailto:m.bussemaker@surrey.ac.uk)

# Reactor dimensions


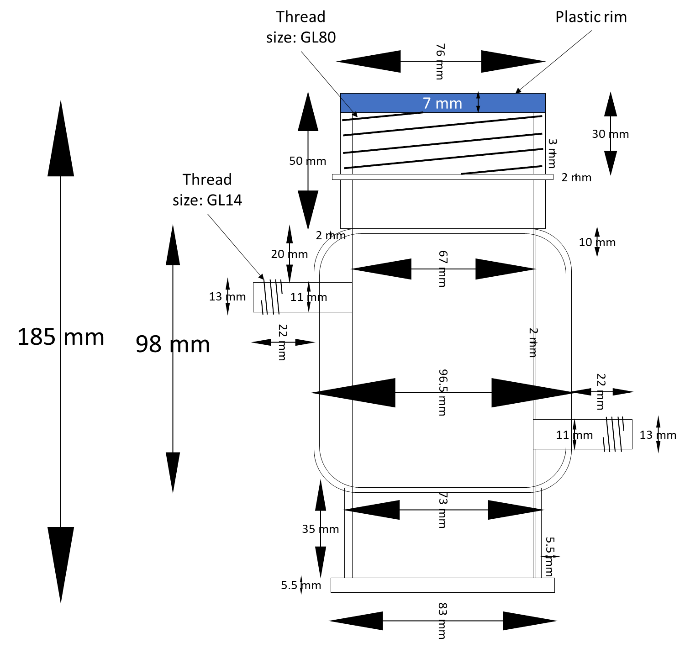

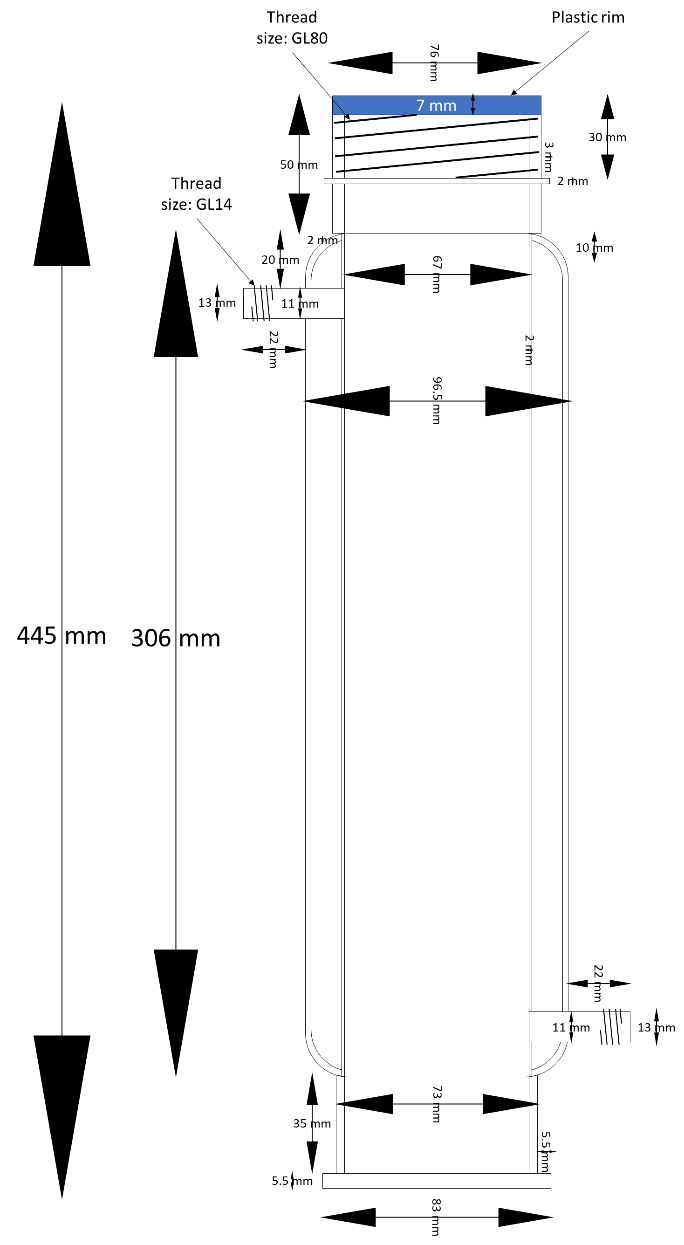


Figure S1: Reactor sizing and designs, 0.6 L reactor (left) and 1.4 L reactor (right)

# Temperatures recorded

Table S1: Temperatures recorded at six different applied powers in a 0.6 L under 410 kHz ultrasound and variable power density with temperature control.

| Liquid height = 113.5 mm, PD_L_ = 100 - 350 W L^-1^, with CW | | | | | | | | | | | | |
| --- | --- | --- | --- | --- | --- | --- | --- | --- | --- | --- | --- | --- |
| Applied power | 40 | | 50 | | 60 | | 80 | | 120 | | 140 | |
| Time (mins) | Av T (°C) | Stand dev | Av T (°C) | Stand dev | Av T (°C) | Stand dev | Av T (°C) | Stand dev | Av T (°C) | Stand dev | Av T (°C) | Stand dev |
| 0 | 25.0 | 1.3 | 21.0 | 0.0 | 21.5 | 0.1 | 22.6 | 0.7 | 20.5 | 0.0 | 19.8 | 0.1 |
| 10 | 25.8 | 1.0 | 24.1 | 0.5 | 26.4 | 0.7 | 26.8 | 1.1 | 34.9 | 0.6 | 38.1 | 1.0 |
| 20 | 24.7 | 1.2 | 25.3 | 0.8 | 27.4 | 0.7 | 27.0 | 1.1 | 38.0 | 0.8 | 42.2 | 1.0 |
| 30 | 24.6 | 1.5 | 25.6 | 0.6 | 27.3 | 0.6 | 26.7 | 0.7 | 38.8 | 1.2 | 43.2 | 1.4 |

Table S2: Temperatures recorded during temperature-controlled and uncontrolled studies at five different liquid heights in a 0.6 L reactor under 410 kHz ultrasound at 100 W L^-1^ and 200 W L^-1^ power density. Average temperatures (Av T) and standard deviations (Stand Dev) are of three repeat experiments.

| PD_L_ = 100 W L^-1^, with CW | | | | | | | | | | |
| --- | --- | --- | --- | --- | --- | --- | --- | --- | --- | --- |
| Liquid height (mm) | 56.7 | | 70.9 | | 85.1 | | 113.5 | | 141.8 | |
| Time (mins) | Av T (°C) | Stand dev | Av T (°C) | Stand dev | Av T (°C) | Stand dev | Av T (°C) | Stand dev | Av T (°C) | Stand dev |
| 0 | 24.2 | 0.7 | 23.8 | 0.9 | 22.9 | 0.4 | 25.0 | 1.3 | 18.0 | 0.4 |
| 5 | 26.4 | 0.8 | 25.7 | 0.6 | 25.9 | 1.1 | 26.3 | 1.0 | 19.3 | 0.9 |
| 10 | 26.2 | 1.3 | 25.9 | 1.2 | 26.4 | 1.4 | 25.0 | 1.0 | 19.5 | 1.3 |
| 15 | 26.2 | 1.1 | 25.6 | 0.9 | 26.2 | 0.8 | 25.2 | 1.7 | 19.0 | 0.9 |
| 20 | 26.0 | 0.4 | 25.2 | 0.4 | 24.7 | 0.7 | 24.7 | 1.2 | 19.2 | 0.6 |
| 25 | 25.2 | 0.3 | 24.7 | 0.2 | 24.4 | 0.4 | 23.6 | 1.0 | 19.3 | 0.5 |
| 30 | 24.6 | 0.4 | 25.6 | 2.2 | 24.2 | 1.3 | 24.6 | 1.5 | 19.4 | 0.7 |
| PD_L_ = 100 W L^-1^, without CW | | | | | | | | | | |
| Liquid height (mm) | 56.7 | | 70.9 | | 85.1 | | 113.5 | | 141.8 | |
| Time (mins) | Av T (°C) | Stand dev | Av T (°C) | Stand dev | Av T (°C) | Stand dev | Av T (°C) | Stand dev | Av T (°C) | Stand dev |
| 0 | 20.1 | 0.1 | 20.9 | 0.1 | 20.9 | 0.1 | 21.3 | 0.0 | 22.1 | 0.1 |
| 5 | 25.6 | 0.3 | 26.4 | 0.4 | 26.6 | 0.3 | 27.2 | 0.6 | 28.1 | 0.6 |
| 10 | 30.1 | 0.7 | 31.1 | 0.6 | 31.3 | 0.7 | 32.1 | 0.9 | 32.7 | 0.7 |
| 15 | 34.2 | 1.0 | 35.0 | 1.0 | 35.5 | 1.1 | 36.5 | 1.2 | 37.2 | 1.3 |
| 20 | 37.5 | 1.0 | 38.4 | 1.0 | 39.1 | 1.2 | 40.1 | 1.3 | 41.2 | 1.6 |
| 25 | 40.3 | 1.1 | 41.8 | 1.3 | 42.4 | 1.5 | 44.0 | 1.2 | Not measured | |
| 30 | 42.7 | 1.0 | 44.4 | 1.5 | 45.1 | 1.5 | 46.4 | 1.6 | 46.9 | 2.0 |
| PD_L_ = 200 W L^-1^, with CW | | | | | | | | | | |
| Liquid height (mm) | 56.7 | | 70.9 | | 85.1 | | 113.5 | | 141.8 | |
| Time (mins) | Av T (°C) | Stand dev | Av T (°C) | Stand dev | Av T (°C) | Stand dev | Av T (°C) | Stand dev | Av T (°C) | Stand dev |
| 0 | 23.5 | 0.5 | 21.8 | 1.1 | 22.3 | 1.8 | 22.6 | 0.7 | 19.2 | 0.2 |
| 5 | 28.5 | 0.9 | 26.1 | 1.1 | 25.5 | 1.3 | 25.9 | 1.1 | 24.1 | 0.3 |
| 10 | 30.4 | 1.2 | 27.5 | 1.4 | 26.8 | 1.8 | 26.8 | 1.1 | 25.7 | 0.4 |
| 15 | 30.9 | 1.4 | 27.4 | 0.9 | 26.6 | 1.4 | 27.1 | 1.3 | 26.5 | 0.5 |
| 20 | 31.0 | 1.0 | 27.6 | 0.9 | 26.8 | 1.5 | 27.0 | 1.1 | 26.3 | 0.0 |
| 25 | 31.1 | 0.9 | 27.6 | 0.7 | 26.8 | 1.3 | 26.8 | 1.0 | 27.3 | 0.7 |
| 30 | 31.1 | 0.6 | 27.7 | 0.6 | 26.8 | 1.1 | 26.7 | 0.7 | 27.3 | 0.8 |
| PD_L_ = 200 W L^-1^, without CW | | | | | | | | | | |
| Liquid height (mm) | 56.7 | | 70.9 | | 85.1 | | 113.5 | | 141.8 | |
| Time (mins) | Av T (°C) | Stand dev | Av T (°C) | Stand dev | Av T (°C) | Stand dev | Av T (°C) | Stand dev | Av T (°C) | Stand dev |
| 0 | 20.5 | 0.5 | 20.5 | 0.1 | 20.2 | 1.9 | 19.6 | 1.6 | 18.5 | 1.7 |
| 5 | 31.3 | 0.4 | 31.8 | 0.1 | 31.7 | 1.8 | 31.8 | 1.4 | 29.7 | 1.7 |
| 10 | 40.0 | 0.2 | 41.1 | 0.1 | 40.2 | 1.5 | 41.8 | 1.7 | 39.5 | 1.4 |
| 15 | 46.9 | 0.2 | 48.8 | 0.1 | 48.7 | 1.4 | 50.6 | 1.4 | 47.9 | 1.2 |
| 20 | 52.4 | 0.1 | 55.0 | 0.1 | 55.8 | 1.3 | 58.0 | 1.3 | 55.1 | 1.2 |
| 25 | 56.6 | 0.0 | 59.9 | 0.1 | 61.6 | 1.2 | 63.5 | 2.1 | 60.6 | 1.3 |
| 30 | 59.9 | 0.1 | 63.8 | 0.0 | 66.2 | 1.1 | 69.3 | 0.9 | 66.0 | 1.1 |

# Use of multiple transducers

Ultrasonic transducers utilise an alternating current (AC) but can be considered like resistors in a direct current (DC) circuit. In an AC circuit, resistance takes on a complex value, impedance (Z, Ohms). Here, $Z_{i}$ will represent the load impedance of a single transducer and $Z_{T}$will represent the total impedance of the load the amplifier supplies to. To power multiple transducers, the amplifier current was split in parallel using a series of T-junction splitters. Load impedances in series add to the total impedance summatively [1], like how adding length to a pipe increases flow resistance. Hence, for $n$ transducers in series, the total series system impedance $(Z_{ST})$is given by Equation S1:

Equation S1

$$Z_{ST}=Z_{1}+Z_{2}+Z_{3}\ldots=\sum_{i=1}^{n} Z_{i}$$

However, adding impedances to an AC circuit in parallel, creates a greater area for electrical flow, in the same way that adding more branches to a pipe would. Hence, impedances in parallel add reciprocally [1] and the total impedance for n parallel transducers ($Z_{PT}$) is given by Equation S2:

Equation S2

$$\frac{1}{Z_{PT}}=\frac{1}{Z_{1}}+\frac{1}{Z_{2}}+\frac{1}{Z_{3}}\ldots+\frac{1}{Z_{n}}$$

Rearranging to get $Z_{PT}$ gives:

Equation S3

$$Z_{PT}=\frac{Z_{1}Z_{2}Z_{3}\ldots Z_{n}}{\frac{Z_{1}Z_{2}Z_{3}\ldots Z_{n}}{Z_{1}}+\frac{Z_{1}Z_{2}Z_{3}\ldots Z_{n}}{Z_{2}}\ldots+\frac{Z_{1}Z_{2}Z_{3}\ldots Z_{n}}{Z_{n}}+}$$

$$={\prod_{i=1}^{n} Z_{i}}/{\sum_{i=1}^{n} \left[ \left( \prod_{i=1}^{n} Z_{i} \right)/{Z_{i}} \right]}$$

For the experimental set up studied here, the transducers are identical and hence the individual impedances are identical. Hence, for $n$ transducers in parallel, Equation S3 can be simplified to Equation S4:

Equation S4

$$Z_{PT}=\frac{{Z_{i}}^{n}}{{{n Z}_{i}}^{n-1}}$$

$$=\frac{Z_{i}}{n}$$

Thus, adding impedances in parallel decreases the total impedance [1]. Impedance matching is an important consideration for effective power transfer in AC electronics, since poor impedance matching generated reflected (and therefore wasted) power [1]. In the system used here, impedence matching was achieved using an SUT.

# Further analytical details

Figure S2: Fluoride probe calibration using PFOS in water, and diluted 3M Lightwater. Error bars represent the standard deviation of three repeats. Trendlines represent only the linear regions.

# Supplementary results

Figure S3: R_F-_ from the sonolysis of 10.0 mg L^-1^ PFOS under 410 kHz ultrasound, in a 0.6 L reactor, at 100 W L^-1^ and 200 W L^-1^ load power, both with and without CW, at five liquid heights. Error bars represent the standard deviation of three repeats.

Table S3: Power Densities (PD), intensities (PI) at various liquid heights and volumes

|  |  | 100 W L^-1^ | | 200 W L^-1^ | |
| --- | --- | --- | --- | --- | --- |
| Liquid height (mm) | Volume (L) | Load power (W) | PI (W cm^-2^) | Load power (W) | PI (W cm^-2^) |
| 26.7 | 0.2 | 20 | 0.57 | 40 | 1.13 |
| 70.9 | 0.25 | 25 | 0.71 | 50 | 1.42 |
| 85.1 | 0.3 | 30 | 0.85 | 60 | 1.7 |
| 113.5 | 0.4 | 40 | 1.13 | 80 | 2.27 |
| 141.8 | 0.5 | 50 | 1.42 | 100 | 2.84 |

Figure S4: R_F-_ from the sonolysis of 10.0 mg L^-1^ PFOS under 410 kHz ultrasound, in a 0.6 L reactor, at 100 W L^-1^ and 200 W L^-1^ load power with cooling water. Plotted as a function of power intensity. Error bars represent the standard deviation of three repeats.

*Figure S5: Sonoluminescence (A) and sonochemiluminescence (B) images for sonolysis of Milli-Q water using 410 kHz ultrasound at 100 W L^-1^ (A-B.1) and 200 W L^-1^ (A-B.2) applied power, in liquid heights of 56.7, 70.9, 85.1, 113.5 and 141.8 mm, respectively. Three repeats are shown, top to bottom of each section. Note the bold shadows seen at the reactor bottom are due to screws and nuts which affix the transducer to the reactor base.*

| **A.1** SL 100 W/L | | | | |
| --- | --- | --- | --- | --- |
| 56.7 mm | 70.9 mm | 85.1 mm | 113.5 mm | 141.8 mm |

































| **A.2** SL 200 W/L | | | | |
| --- | --- | --- | --- | --- |
| 56.7 mm | 70.9 mm | 85.1 mm | 113.5 mm | 141.8 mm |

































| **B.1** SCL 100 W/L | | | | | | | | | |
| --- | --- | --- | --- | --- | --- | --- | --- | --- | --- |
| 56.7 mm | | 70.9 mm | | 85.1 mm | | 113.5 mm | | 141.8 mm | |
|  | |  | |  | |  | |  | |
|  | |  | |  | |  | |  | |
|  | |  | |  | |  | |  | |
| **B.2** SCL 200 W/L | | | | | | | | |  |
| 56.7 mm | 70.9 mm | | 85.1 mm | | 113.5 mm | | 141.8 mm | |  |

































| **A.2** SL 200 W/L | | | | |
| --- | --- | --- | --- | --- |
| 56.7 mm | 70.9 mm | 85.1 mm | 113.5 mm | 141.8 mm |

































| **B.1** SCL 100 W/L | | | | | |
| --- | --- | --- | --- | --- | --- |
| 56.7 mm | 70.9 mm | 85.1 mm | 113.5 mm | 141.8 mm | |
|  |  |  |  |  | |
|  |  |  |  |  | |
|  |  |  |  |  | |
|  | | | | |  |

| **B.2** SCL 200 W/L | | | | |
| --- | --- | --- | --- | --- |
| 56.7 mm | 70.9 mm | 85.1 mm | 113.5 mm | 141.8 mm |



















# Fluoride Release Rate Data

Table S4: Measured fluoride concentrations over 30 min at different liquid heights with constant power densities. in a 0.6 L reactor under 410 kHz ultrasound at 100 W L^-1^ and 200 W L^-1^ power density. Average concentrations and standard deviations (SD) are of three repeat experiments.

| PD_L_ = 100 W L^-1^, with CW | | | | | | | | | | |
| --- | --- | --- | --- | --- | --- | --- | --- | --- | --- | --- |
| Liquid height (mm) | 56.7 | | 70.9 | | 85.1 | | 113.5 | | 141.8 | |
| Time (mins) | Av F^-^ Conc (μM) | SD | Av F^-^ Conc (μM) | SD | Av F^-^ Conc (μM) | SD | Av F^-^ Conc (μM) | SD | Av F^-^ Conc (μM) | SD |
| 5 | 4.36 | 0.28 | 3.48 | 0.07 | 3.33 | 0.15 | 3.64 | 0.38 | 3.50 | 0.30 |
| 10 | 9.54 | 0.06 | 8.48 | 0.10 | 8.04 | 0.27 | 8.27 | 0.86 | 8.50 | 0.56 |
| 15 | 14.96 | 0.88 | 13.93 | 0.31 | 13.27 | 0.41 | 13.83 | 0.99 | 14.65 | 0.60 |
| 20 | 21.30 | 0.72 | 19.92 | 0.42 | 18.96 | 0.65 | 19.87 | 1.32 | 20.89 | 0.43 |
| 25 | 27.06 | 1.23 | 25.98 | 0.76 | 25.41 | 0.23 | 26.72 | 0.90 | 28.54 | 1.35 |
| 30 | 33.43 | 0.92 | 32.65 | 0.96 | 31.90 | 0.13 | 34.05 | 0.42 | 37.50 | 0.98 |
| PD_L_ = 200 W L^-1^, with CW | | | | | | | | | | |
| Liquid height (mm) | 56.7 | | 70.9 | | 85.1 | | 113.5 | | 141.8 | |
| Time (mins) | Av F^-^ Conc (μM) | SD | Av F^-^ Conc (μM) | SD | Av F^-^ Conc (μM) | SD | Av F^-^ Conc (μM) | SD | Av F^-^ Conc (μM) | SD |
| 5 | 11.15 | 0.20 | 12.08 | 1.07 | 13.68 | 1.97 | 12.51 | 1.00 | 12.42 | 3.52 |
| 10 | 25.50 | 0.11 | 27.60 | 2.08 | 31.78 | 4.02 | 29.38 | 1.22 | 28.94 | 6.76 |
| 15 | 41.43 | 0.92 | 40.61 | 8.91 | 48.47 | 2.21 | 47.04 | 2.13 | 47.34 | 8.94 |
| 20 | 57.70 | 2.26 | 61.85 | 4.65 | 65.62 | 2.89 | 64.48 | 3.23 | 65.00 | 9.59 |
| 25 | 74.07 | 3.79 | 79.35 | 4.73 | 82.59 | 4.72 | 82.95 | 3.91 | 80.87 | 6.71 |
| 30 | 90.00 | 5.39 | 96.78 | 5.77 | 99.99 | 4.27 | 100.10 | 5.24 | 96.38 | 3.37 |

Table S5: Measured fluoride concentrations from sonication of different AFFF dilutions. Flow-though conditions, at 214.2 ml min^-1^ recirculation rate; 3 x 500 mL was used with 270 W L^-1^ ultrasound at 410 kHz, 3 x parallel reactors.

| Dilution factor | 5× | | 20× | |
| --- | --- | --- | --- | --- |
| Time (mins) | Av F^-^ Conc (μM) | SD | Av F^-^ Conc (μM) | SD |
| 30 | 12.17 | 1.15 | 107.6 | 5.3 |
| 60 | 27.09 | 5.00 | 246.9 | 2.0 |
| 90 | 32.39 | 4.44 | 404.1 | 4.5 |
| 120 | 67.68 | 1.11 | 508.9 | 1.7 |
| Dilution factor | 10× | |  |  |
| Time (mins) | Av F^-^ Conc (μM) | SD |  |  |
| 120 | 427.2 | 15.2 |  |  |
| 240 | 639.9 | 27.8 |  |  |
| 360 | 886.1 | 24.2 |  |  |
| 480 | 1351.6 | 8.1 |  |  |
| Dilution factor | 100× | |  |  |
| Time (mins) | Av F^-^ Conc (μM) | SD |  |  |
| 60 | 42.0 | 9.4 |  |  |
| 120 | 39.5 | 18.8 |  |  |
| 150 | 104.8 | 7.1 |  |  |
| 180 | 115.6 | 26.6 |  |  |
| 210 | 136.7 | 5.2 |  |  |
| 240 | 202.9 | 11.4 |  |  |
| 300 | 234.3 | 14.0 |  |  |
| 420 | 312.9 | 18.8 |  |  |
| 480 | 354.8 | 22.6 |  |  |
